# A Novel Broadband Forcecardiography Sensor for Simultaneous Monitoring of Respiration, Infrasonic Cardiac Vibrations and Heart Sounds

**DOI:** 10.3389/fphys.2021.725716

**Published:** 2021-11-18

**Authors:** Emilio Andreozzi, Gaetano D. Gargiulo, Daniele Esposito, Paolo Bifulco

**Affiliations:** ^1^Department of Electrical Engineering and Information Technologies, University of Naples Federico II, Naples, Italy; ^2^School of Engineering, Design and Built Environment, Western Sydney University, Penrith, NSW, Australia

**Keywords:** forcecardiography, seismocardiography, heart sounds, respiration, heart vibrations, piezoelectric sensor, cardiopulmonary monitoring, phonocardiography

## Abstract

The precordial mechanical vibrations generated by cardiac contractions have a rich frequency spectrum. While the lowest frequencies can be palpated, the higher infrasonic frequencies are usually captured by the seismocardiogram (SCG) signal and the audible ones correspond to heart sounds. Forcecardiography (FCG) is a non-invasive technique that measures these vibrations via force sensing resistors (FSR). This study presents a new piezoelectric sensor able to record all heart vibrations simultaneously, as well as a respiration signal. The new sensor was compared to the FSR-based one to assess its suitability for FCG. An electrocardiogram (ECG) lead and a signal from an electro-resistive respiration band (ERB) were synchronously acquired as references on six healthy volunteers (4 males, 2 females) at rest. The raw signals from the piezoelectric and the FSR-based sensors turned out to be very similar. The raw signals were divided into four components: Forcerespirogram (FRG), Low-Frequency FCG (LF-FCG), High-Frequency FCG (HF-FCG) and heart sounds (HS-FCG). A beat-by-beat comparison of FCG and ECG signals was carried out by means of regression, correlation and Bland–Altman analyses, and similarly for respiration signals (FRG and ERB). The results showed that the infrasonic FCG components are strongly related to the cardiac cycle (*R*^2^ > 0.999, null bias and Limits of Agreement (LoA) of ± 4.9 ms for HF-FCG; *R*^2^ > 0.99, null bias and LoA of ± 26.9 ms for LF-FCG) and the FRG inter-breath intervals are consistent with ERB ones (*R*^2^ > 0.99, non-significant bias and LoA of ± 0.46 s). Furthermore, the piezoelectric sensor was tested against an accelerometer and an electronic stethoscope: synchronous acquisitions were performed to quantify the similarity between the signals. ECG-triggered ensemble averages (synchronized with R-peaks) of HF-FCG and SCG showed a correlation greater than 0.81, while those of HS-FCG and PCG scored a correlation greater than 0.85. The piezoelectric sensor demonstrated superior performances as compared to the FSR, providing more accurate, beat-by-beat measurements. This is the first time that a single piezoelectric sensor demonstrated the ability to simultaneously capture respiration, heart sounds, an SCG-like signal (i.e., HF-FCG) and the LF-FCG signal, which may provide information on ventricular emptying and filling events. According to these preliminary results the novel piezoelectric FCG sensor stands as a promising device for accurate, unobtrusive, long-term monitoring of cardiorespiratory functions and paves the way for a wide range of potential applications, both in the research and clinical fields. However, these results should be confirmed by further analyses on a larger cohort of subjects, possibly including also pathological patients.

## Introduction

Cardiac monitoring has always been a critical task in medicine, all the more so if one considers the current burden of cardiovascular diseases (Gersh et al., 2010; Benjamin et al., 2018). Undoubtedly, palpation and auscultation have been the first monitoring techniques adopted by healers and physicians, which provided information about the mechanical behavior of the heart. From the end of the XIX century, scientific researchers have investigated several objective methods to gain further insights into the mechanical events that occur within the cardiac cycle, both in healthy and pathological subjects (Knoop, 1965; Luisada et al., 1985; Zanetti and Salerno, 1990). These methods were based on various kinds of mechanical sensors and well-known examples are Phonocardiography (PCG) (Rappaport and Sprague, 1942; Ismail et al., 2018), Kinetocardiography (Eddleman et al., 1953), Apexcardiography (Marey, 1878; Benchimol and Dimond, 1962), Dynamocardiography (Komarov, 1958; Babskiy and Karpman, 1964), Ballistocardiography (BCG) (Gordon, 1877; Burger and Noordergraaf, 1956; Starr, 1958; Knoop, 1965; Sadek et al., 2019), and Seismocardiography (SCG) (Zanetti and Salerno, 1990; Inan et al., 2015; Taebi et al., 2019). These techniques have been the only means available to researchers to inspect the mechanical behavior of the heart non-invasively, until the rise of ultrasound investigations have provided the ability to actually see the movements of internal body parts with unprecedented accuracy in real time. Indeed, due to the cumbersome instrumentation involved and the uneasiness of signals interpretation, these techniques lost their appeal for both research and clinical purposes (Zanetti and Salerno, 1990).

In the last decades, the technological advances made miniaturized, lightweight and unobtrusive sensors available, especially accelerometers based on microelectromechanical systems (MEMS) technology, therefore, some of those techniques, namely SCG and BCG, started gaining progressively more appeal as a mean to enable wearable applications for long-term, continuous monitoring (Zanetti and Salerno, 1990; Inan et al., 2015). Indeed, one of the current limitations of ultrasound imaging technology is being unsuitable to wearable applications and pervasive monitoring, rather standing as an invaluable clinical tool for accurate diagnosis.

Along this new path, many possibilities have been examined (Inan et al., 2015; Taebi et al., 2019). Paukkunen et al. (2016) explored the SCG analysis based on triaxial accelerations in place of the typical dorso-ventral acceleration alone. The significance of SCG has been further explored via comparison with echocardiography and also with other measurements modalities, such as PCG and impedance cardiography, in order to better understand its relationship with the events of the cardiac cycle (Crow et al., 1994; Leitão et al., 2018; Lin et al., 2018; Dehkordi et al., 2019). These studies contributed to the improvement of SCG annotation and have also led to the definition of novel fiducial points (Taebi et al., 2019), such as peaks of heart walls velocities and peaks of blood flow through the heart valves (Lin et al., 2018). Deeper investigation on the origin of these fiducial points has been carried out via modeling and simulation approaches (Gurev et al., 2012), also in BCG research (Kim et al., 2016). Concerning the annotation task, various approaches have been proposed to improve its accuracy and robustness (Khosrow-Khavar et al., 2017; Choudhary et al., 2018a; Sørensen et al., 2018; Mora et al., 2019) to morphological alterations, motion artifacts and noise, which have indeed represented the main weaknesses of SCG since its first introduction in the scientific community (Zanetti and Salerno, 1990; Inan et al., 2015; Taebi et al., 2019). Particular effort has been devoted to methods for motion artifacts removal, e.g., via normalized least mean square adaptive filters (Yang and Tavassolian, 2016), independent component analysis (Yang and Tavassolian, 2018a), empirical mode decomposition (Javaid et al., 2017; Taebi and Mansy, 2017), adaptive recursive least square filters (Yu and Liu, 2020), and also by using two triaxial accelerometers (Luu and Dinh, 2018). In addition, a quality index for the automatic recognition of severely corrupted segments has been developed (Zia et al., 2019), as well as a method to recognize signals provided by misplaced sensors (Ashouri and Inan, 2017). These solutions both help discard inaccurate signals from SCG analysis, so as to ensure reliable diagnostic results, and represent another step toward a trustworthy, SCG-based monitoring in unsupervised settings. Recently, also the well-known intra-subject and inter-subject variability of SCG morphology has been addressed for the purpose of improving the measurement accuracy of time intervals within the cardiac cycles (e.g., the pre-ejection period) (Zia et al., 2020a,b).

A relevant trend in SCG research have focused on the extraction of well-established physiological signals from SCG recordings, such as respiration signals (Pandia et al., 2012; Jafari Tadi et al., 2014; Azad et al., 2018) and heart sounds (Castiglioni et al., 2011; Jain et al., 2016; Choudhary et al., 2018b). The respiratory information is usually extracted by estimating either the baseline wandering of the acceleration signals (Jafari Tadi et al., 2014), or the amplitude modulation of the SCG waves corresponding to each heartbeat (Pandia et al., 2012; Azad et al., 2018).

Novel sensors, instruments and techniques, both contact-based and contactless, have been proposed to measure the cardiac-induced vibrations of the chest wall non-invasively (Taebi et al., 2019). Among the contact-based approaches, the use of gyroscopes has been investigated for the measurement of the rotational components of chest wall motion, which gave birth to the Gyrocardiography (GCG) technique (Jafari Tadi et al., 2017; Dehkordi et al., 2020; Sieciński et al., 2020). The performances of gyroscopes have also been evaluated in combination with triaxial accelerometers (Yang et al., 2017; Yang and Tavassolian, 2018b; D’Mello et al., 2019; Shandhi et al., 2019). In particular, the novel Kinocardiography (KCG) technique has been proposed, which is based on two inertial measurement units, placed onto the chest and on the lumbar region, each one equipped with a 3-axis accelerometer and a 3-axis gyroscope. The SCG and GCG signals thus acquired are used to compute the integral of kinetic energy, both for the linear and rotational components of body motion, which can be used to monitor the cardiac inotropic activity (Hossein et al., 2021a,b). Since it has been for long recognized that heart-induced vibrations measured at different sites of the chest wall show morphological differences, novel multisite measurement systems have been developed (Lin et al., 2018; Di Rienzo et al., 2020; Munck et al., 2020). In particular, a multichannel SCG system based on a 4-by-4 matrix of triaxial accelerometers has been proposed by Munck et al. (2020) The system allows obtaining surface vibration maps via simultaneous, multisite measurements of chest wall vibrations, thus standing as a novel kind of imaging modality for the analysis of the mechanical behavior of the beating heart (Munck et al., 2020). In addition to MEMS sensors, also piezoelectric sensors have been investigated for SCG recording (Bifulco et al., 2014; Ha et al., 2019; Nayeem et al., 2020). Very recently, the novel Forcecardiography (FCG) technique has been proposed (Andreozzi et al., 2020, 2021). FCG is based on the use of custom-designed force sensors (FCG sensors) that allow measuring the heart-induced vibrations of chest wall (Andreozzi et al., 2020) and the respiratory activity simultaneously (Andreozzi et al., 2021), thus standing as a novel promising technique for cardiorespiratory monitoring. FCG sensors are based on force sensitive resistors (FSR) that have already proved viable for muscle contraction monitoring (Esposito et al., 2018) and hand gestures recognition (Esposito et al., 2020). FCG proved capable of acquiring, in addition to a Seismocardiogram-like signal, (referred to as HF-FCG) that could provide information on opening and closure of heart valves (Zanetti and Salerno, 1990; Inan et al., 2015; Taebi et al., 2019) a novel low-frequency component (referred to as LF-FCG) that seems to carry information on ventricular filling and emptying dynamics and cannot be appreciated from common SCG recordings (Andreozzi et al., 2020).

An interesting trend concentrated on the development of integrated sensors systems for unobtrusive multimodal sensing. Indeed, in many researches the need to simultaneously acquire multiple physiological signals has been addressed by placing different sensors onto each subject, which usually makes the measurements uncomfortable, all the more so when long-term monitoring is to be performed. Various integrated sensors systems have been proposed, which can be attached to the subject’s chest and are based on small printed circuit boards equipped with MEMS accelerometers and the electronics for SCG and ECG acquisition (Chuo et al., 2009; Leitão et al., 2018; Shandhi et al., 2020). In addition, devices capable of measuring also acoustic signals have been introduced. Bifulco et al. (2014) proposed a polyvinylidenefluoride (PVDF) sensor that provides a signal containing respiration, SCG and heart sounds components. A PVDF-based, ultrathin, stretchable, E-tattoo sensor has been presented, which allows acquiring ECG, SCG and heart sounds simultaneously (Ha et al., 2019). Similarly to the E-tattoo, an epidermal mechano-acoustic sensor based on soft electronics has been proposed (Liu et al., 2016). The device is equipped with a triaxial accelerometer and dry electrodes for simultaneous mechano-acoustic and electrophysiological sensing. Being very small and flexible, the sensor easily adapts to curved skin surfaces, where common rigid sensors platforms would hardly achieve a stable mechanic coupling. More recently, some of the authors of Liu et al. (2016) presented a novel skin-compliant device to be placed at the suprasternal notch, from which it proved capable of monitoring various activities such as respiration, cardiac activity, swallowing, vocal-fold vibrations, locomotion and body orientation, by means of triaxial accelerometers with an 800 Hz bandwidth (Lee et al., 2019). Another recent approach focused on the development of a monolithic integrated sensor system for multimodal sensing of cardiopulmonary signals. The device comprises a triaxial accelerometer for monitoring of body motion, respiration and SCG, and a piezoelectric sensor, for simultaneous recording of heart and lung sounds (Gupta et al., 2020).

These recent trends clarify that a thorough assessment of cardiorespiratory functions requires the recording of multiple physiological signals, obtained via multiple sensors mounted onto the monitored subject or, according to the most recent technological advancements, via *ad hoc* designed devices obtained by integrating multiple sensors (e.g., accelerometers, gyroscopes, piezoelectric/acoustic sensors, ECG electrodes).

FCG sensors have already proved capable of providing accurate measurement of heart and respiration rates, besides capturing novel information about the heart contraction. In this study, a novel FCG sensor was investigated, which is capable of capturing also the heart sounds, thus enriching the information that could be acquired via the FCG technique. In particular, a novel FCG sensor based on a small piezoelectric lead-zirconate-titanate (PZT) disk is demonstrated to be a suitable device for multimodal cardiorespiratory sensing, offering the opportunity to monitor respiration, heart sounds, seismocardiogram and the potentially ventricular-volume-related signal (i.e., the LF-FCG), simultaneously from one single signal: the forcecardiogram. The piezoelectric sensor was first compared, in terms of signal morphology, with the FSR-based sensor that had been originally employed to develop the FCG technique (Andreozzi et al., 2020, 2021). Then, the cardiorespiratory monitoring performances of the piezoelectric sensor were assessed via comparison with a respiratory electro-resistive band (ERB) (Jayarathna et al., 2020) and an ECG monitor, considered as benchmarks for respiratory and heart rate measurement, respectively.

## Materials and Methods

### Forcecardiography Sensors

In this study, a novel piezoelectric sensor was proposed to acquire FCG signals. To prove its suitability for FCG, it was compared to the first, FSR-based sensor presented for FCG measurement in Andreozzi et al. (2020).

In particular, the FSR-based sensor adopted in this study comprised an Ohmite FSR03CE (Ohmite Mfg Co., Warrenville, IL, United States), which has an external diameter of 30.50 mm and an active area of 25.42 mm. The FSR was equipped with a dome-shaped mechanical coupler, so as to ensure a good transduction of the force to its active area. Considering the linear relationship between the applied force and the FSR electrical conductance, a conditioning circuit based on a transimpedance amplifier was used, which ensured high linearity while minimizing the sensor drift by keeping the voltage across the FSR at a constant value (Paredes-Madrid et al., 2017a,b; Esposito et al., 2018; Andreozzi et al., 2020, 2021).

**FIGURE 1:**
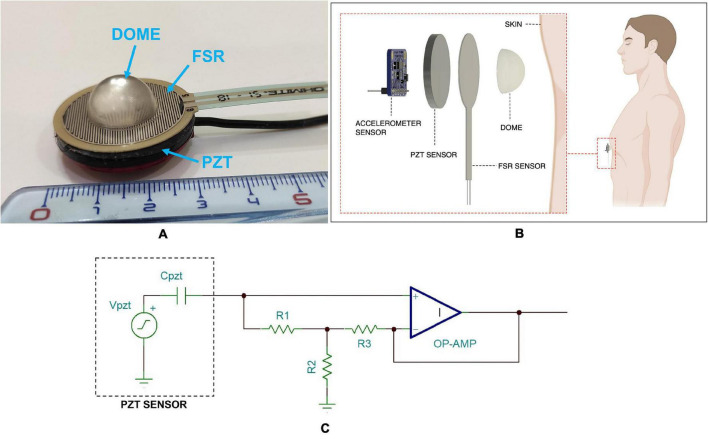
**(A)** The FCG sensors assembly with FSR attached onto the PZT sensor and dome-shaped mechanical coupler; **(B)** schematic representation of the FCG sensors assembly as applied to a subject’s skin; **(C)** schematic of the conditioning circuit for the PZT sensor.

The novel piezoelectric sensor proposed for FCG measurement is a lead-zirconate-titanate (PZT) piezoelectric disk equipped with the same dome-shaped mechanical coupler used for the FSR-based sensor. The PZT sensor has the same external diameter as the Ohmite FSR03CE and an electrical capacitance of 22 nF (measured at 2 kHz via a GWINSTEK LCR-816 LCR meter). A conditioning circuit based on a simple voltage amplifier was adopted (see Figure 1C), which featured a good response at low frequencies (cut-off frequency < 0.005 Hz), in order to preserve also the respiration-related components.

For comparison purposes, the FSR, equipped with the dome-shaped mechanical coupler, was attached onto the PZT sensor, so as to allow simultaneous acquisitions of both FSR and PZT signals from the same point onto the chest wall (Gargiulo et al., 2021). The FCG sensors assembly is depicted in Figure 1A, while Figure 1B shows a schematic representation of the assembly as applied to a subject’s skin.

### Electro-Resistive Respiration Band

The assessment of FCG sensor performances in respiration monitoring required the comparison with a reference method. The respiration monitoring method presented in Jayarathna et al. (2020), which is based on the use of an ERB applied on the chest of the subject, was adopted as a benchmark (Andreozzi et al., 2021). An ERB consists of a stretchable stripe or cord, made of conductive rubber, that increases its electrical resistance when stretched. Hence, it can be used to monitor the increase and decrease of chest circumference that occur during the inhalation and exhalation phases of the respiratory acts.

### Measurement Setup and Protocol

**FIGURE 2:**
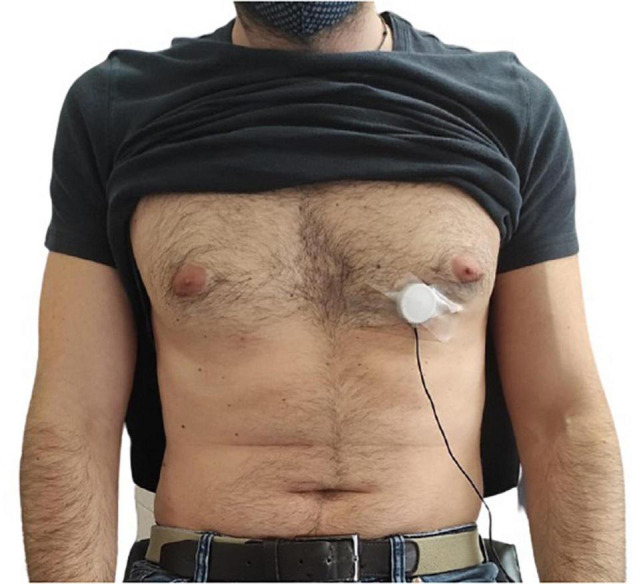
Example of FCG sensor placement onto the chest of a subject by means of medical adhesive tape.

The FCG sensors assembly was placed onto the chest of each subject via a medical adhesive tape, by roughly locating the point of maximal impulse (PMI), and then fastened with a belt around the thorax (see Figure 2). To this aim, multiple contact points on the chest were tested around the point on the fifth intercostal space on the midclavicular line (i.e., the common location of the PMI), and the point corresponding to the maximum signal amplitude was selected for each subject. In order to avoid interference with the FCG sensors assembly, the ERB was mounted onto the upper chest of the subject. An ECG lead I was also acquired by means of a WelchAllyn Propaq ^®^ Encore monitor (Welch Allyn Inc., New York, NY, United States). Simultaneous measurement with an accelerometer and an electronic stethoscope were carried out. A Freescale MMA7361 accelerometer was fixed on the PZT sensor (as in Andreozzi et al., 2020) and the z-axis acceleration was recorded to acquire the dorso-ventral SCG. Furthermore, an Aethra Telestethphone electronic stethoscope, made of a Littman chest-piece and a part of the tubing coupled with a microphone, was placed onto subjects’ chest as close as possible to the PZT sensor, to record a reference PCG signal. The signals from the sensors and the ECG monitor were simultaneously acquired via a National Instrument NI-USB4431 DAQ board, with 24-bit precision and 10 kHz sampling frequency.

Six healthy volunteers (4 males, 2 females, age 36.6 ± 11.0), who signed the informed consent, were enrolled for the experiments. They were asked to comfortably sit on a chair, leaning against the seatback while keeping their back straight. Multiple acquisitions were performed for each subject, who was asked to breath at a variable respiratory pace, so as to obtain measurements of breathing and heart rates in reasonably wide ranges at rest. The subjects were also asked to hold their breath (apnea) while performing the simultaneous recording with the accelerometer and the electronic stethoscope.

### Signal Processing and Analysis

#### Pre-processing and Correlation Analysis of Force Sensing Resistors and Piezoelectric Sensors Signals

The raw signals measured by the FSR-based and PZT sensors contained components related to both respiratory and cardiac activities, which were analyzed independently. To this aim, the raw signals were pre-processed in order to separate these components. All processing and analyses were carried out in MATLAB ^®^ R2017b (The MathWorks, Inc., 1 Apple Hill Drive, Natick, Massachusetts, 01760, United States). The respiratory component, referred to as Forcerespirography (FRG), was first extracted from the raw signals via a 3rd order Savitzki-Golay filter (Savitzky and Golay, 1964), with a frame length corresponding to about a 1.5 s interval, and then subtracted from the raw signal to isolate the FCG component. This procedure allows preserving the actual shape of the FRG component, as compared to a simple low-pass filtering, which could cut off potential high-frequency components (especially in case of forceful inspirations/expirations), also causing artifacts in the resulting FCG signal. The same processing was applied to the signal provided by the ERB.

To prove that the novel PZT sensor is suitable for FCG, the similarity of the ECG-triggered ensemble averages (synchronized with R-peaks) of the two FCG signals obtained from the FSR-based and PZT sensors was assessed by evaluating their correlation. In particular, first, the absolute maximum of their cross-correlation function was located to determine their time lag, which was used for time alignment; then their Pearson’s correlation coefficient was computed to evaluate their similarity.

Afterward, each FCG signal was split into three components for further analyses, namely low-frequency FCG (LF-FCG), high-frequency FCG (HF-FCG) and heart sounds FCG (HS-FCG), by means of 2nd order Butterworth band-pass filters with cut-off frequencies set at 0.5–5 Hz, 7–30 Hz, and 30–200 Hz, respectively.

#### Statistical Analyses

The performances of the novel piezoelectric FCG sensor for respiration and cardiac monitoring were assessed by evaluating its ability to detect the respiratory acts and the heartbeats, as well as the accuracy and precision of the derived respiratory and heart rate measurement.

The respiration monitoring performances were assessed by assuming the ERB signal as the reference. The positive, inspiratory peaks were located via the MATLAB ^®^ function “findpeaks” (basically, this function finds the local maxima in an array; more details about this function are freely available at the Mathworks website.) in both the ERB and the FRG signals that had been acquired simultaneously, then the missed and spurious peaks in the FRG signal were detected by comparison with the ERB signal. The inter-breath intervals were computed and those related to the missed and spurious peaks were excluded from the following analyses. Regression, correlation and Bland-Altman analyses were carried out via the MATLAB ^®^ function “bland-altman-and-correlation-plot” (Ran, 2020) to compare the inter-breath intervals obtained from the FRG and the ERB signals.

The performances for cardiac monitoring were assessed with a similar procedure, by considering the actual FCG signals provided by the PZT sensor and assuming the ECG as the reference. To this aim, the R-peaks first were located in the ECG signal via the well-known Pan and Thompkins algorithm, implemented in the “*BioSigKit*” MATLAB ^®^ toolbox (Sedghamiz, 2018). Then, the heartbeats were located both in the LF-FCG and HF-FCG signals and analyzed separately. In the LF-FCG component, two fiducial markers were considered, namely the typical negative peak that usually appears about the end of the ECG T-wave (Andreozzi et al., 2020) and the negative peak of the first derivative of the LF-FCG signal (i.e., its first-order forward finite difference). In the HF-FCG component, which is an SCG-like signal (Andreozzi et al., 2020), the first positive peak after the related R-peak was considered as a fiducial marker. The detection of heartbeats in the FCG signals took advantage of the *a priori* knowledge of R-peaks locations. The missed heartbeats detected in the FCG signals by comparison with the ECG were annotated; the inter-beat intervals were computed from both the ECG R-peaks and the FCG fiducial markers and those related to the missed peaks were excluded from the following regression, correlation and Bland-Altman analyses.

#### Comparison With Seismocardiography and Phonocardiography

A morphological comparison between HF-FCG and SCG, and between HS-FCG and PCG, was carried out to quantify the similarity of these signals. To this aim, the acceleration and PCG signals were pre-processed via the same filters used to extract the HF-FCG and HS-FCG, respectively. Then, the ECG-triggered ensemble averages (synchronized with R-peaks) of the HF-FCG, HS-FCG, SCG and PCG signals were computed and, finally, the normalized cross-correlation indices of the ensemble averages of HF-FCG vs. SCG and HS-FCG vs. PCG were evaluated for each subject. A more in-depth analysis of HF-FCG and SCG signals similarity was carried out by computing the cross-correlation index of their ensemble averages separately in three distinct intervals, namely systolic, diastolic, and end-diastolic. The systolic interval was defined as a 200-ms interval starting from the ECG Q wave, the diastolic as the interval starting from 50 ms before the “aortic closure” (AC) marker of accelerometric SCG and ending at 150 ms after AC, the end-diastolic as a 200-ms interval ending at the ECG Q wave.

## Results

### Comparison of Forcecardiography Signals From Force Sensing Resistor and Piezoelectric Sensors

**FIGURE 3:**
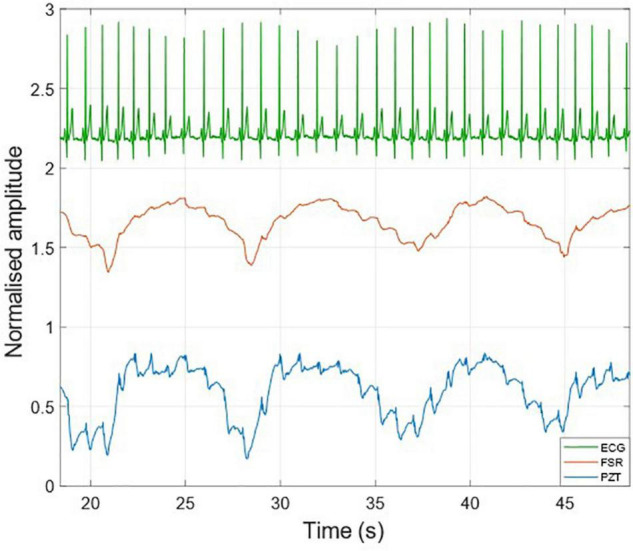
Raw signals acquired from PZT (blue line) and FSR (red line) sensors with simultaneously acquired ECG (green line).

**FIGURE 4:**
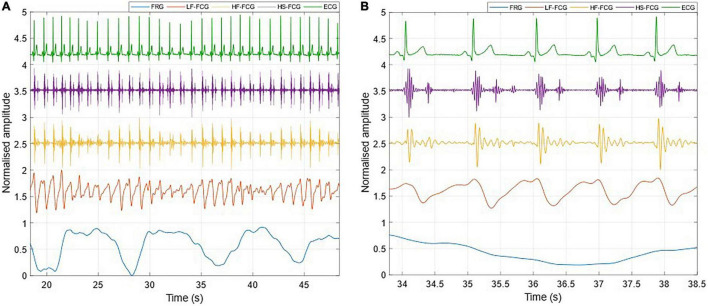
All components extracted from the FCG signal acquired by the PZT sensor. **(A)** 30-s excerpt from subject #2; **(B)** 5-beats excerpt at the beginning of **(A)**.

**FIGURE 5:**
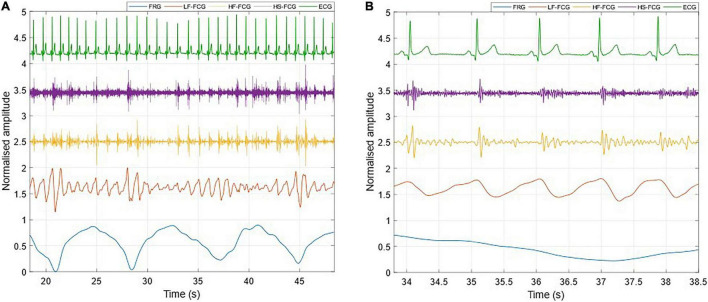
All components extracted from the FCG signal acquired by the FSR-based sensor. **(A)** 30-s excerpt from subject #2; **(B)** 5-beats excerpt from **(A)**.

Figure 3 shows an example of the raw signals acquired simultaneously by the PZT and the FSR-based sensors, together with the ECG. It can be observed that both sensors captured the large, low-frequency components related to the respiratory activity and the smaller FCG component due to the cardiac activity. In Figures 4, 5, the respiratory component (FRG) and the three FCG components (LF-FCG, HF-FCG, HS-FCG) are depicted, which have been extracted from the PZT and FSR-based sensors signals shown in Figure 3. By comparing Figures 4, 5, one can observe that the novel piezoelectric FCG sensor provides more accurate measurements of the HF-FCG component, as well as very clear recordings of heart sounds, as opposed to those provided by the FSR-based sensor, which exhibit a considerably lower signal-to-noise ratio (SNR). Moreover, different amplitude ratios between cardiac and respiratory components can be observed in the PZT and FSR-based sensors signals. Indeed, in the PZT sensor signals, the peak-to-peak amplitudes of LF-FCG, HF-FCG and HS-FCG turned out to be about 24, 9, and 2% of the FRG amplitude, respectively; in the FSR-based sensor signals, instead, these amplitude ratios turned out to be about 12, 3, and 1%, respectively.

**FIGURE 6:**
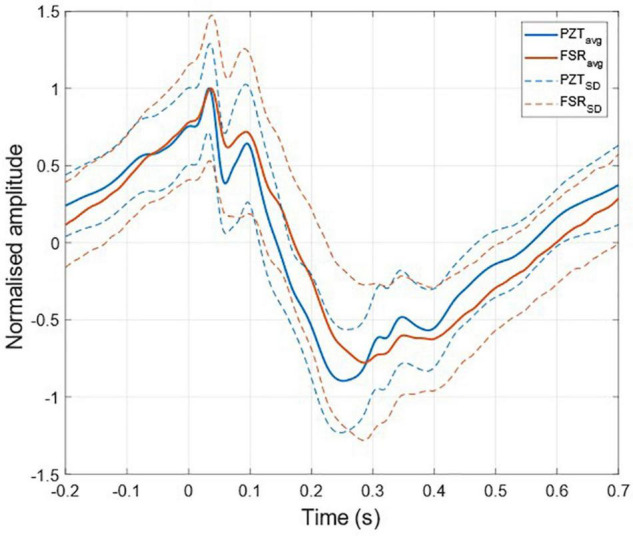
ECG-triggered ensemble averages of FCG signals from FSR and PZT sensors computed on 220 heartbeats. The time 0 corresponds to the location of the ECG R-peak. Ensemble averages are depicted with solid lines, while the limits of the ± SD ranges are depicted with dotted lines. For each sensor signal, the ensemble average and the limits of the ± SD ranges were normalized to the maximum of the ensemble average.

The ECG-triggered ensemble averages (computed on 220 heartbeats) of the FCG components from the PZT and FSR-based sensors signals of subject #3 are shown in Figure 6 (solid lines), along with their standard deviation (SD) ranges (dotted lines). The analysis of their cross-correlation function revealed a time lag of 18.6 ms of the FSR signal with respect to the PZT signal. The two ensemble averages were re-aligned using the computed lag and their Pearson’s correlation coefficient was computed, which turned out to be 0.972 (*p* < 0.05). Such a high similarity confirmed that the novel PZT sensor is suitable for FCG measurement. Moreover, it can be noted that the SD ranges of the PZT sensor signal are narrower with respect to the signal from the FSR-based sensor. This reduction in the variability of FCG signal morphology across different heartbeats, suggests that the novel piezoelectric FCG sensor ensures more precise and stable measurements as compared to the FSR-based one, as previously noticed with regard to the HF-FCG and HS-FCG components from PZT and FSR-based sensors shown in Figures 4, 5.

### Statistical Analyses of Respiration Monitoring

**FIGURE 7:**
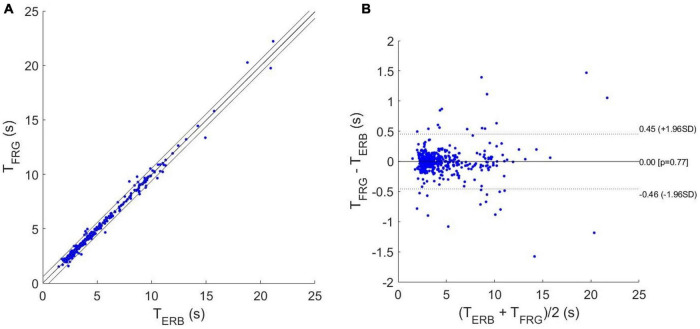
Statistical analyses on inter-breath intervals estimated from ERB and FRG: **(A)** results of regression and correlation analyses; **(B)** results of Bland-Altman analysis.

In Table 1, the number of respiratory acts detected per subject in the ERB and FRG signals are reported, along with the number of missed and spurious acts identified in the latter. In particular, a total of 527 respiratory acts were detected in the ERB signal; only 2 missed peaks were found in the FRG signal, along with 12 spurious peaks that were misclassified as actual respiratory acts. Therefore, the FRG scored a sensitivity of 99.6% and a positive predictive value (PPV) of 97.8%. The inter-breath intervals related to the respiratory acts detected in the ERB and FRG signals were further compared via regression, correlation and Bland-Altman analyses (Figure 7). To this aim, the intervals related to the spurious respiratory acts were discarded from both the FRG and ERB signals, in order to carry out the analyses only on reliable measurements. The statistical analyses were performed on 516 inter-breath intervals and reported a slope and intercept of 0.996 and 0.0129 s, with an *R*^2^-value in excess of 0.992, as well as a non-significant bias (*p* = 0.77) with limits of agreement (LoA) of ± 0.455 s.

**TABLE 1:** Respiration acts detected in the ERB and in the FRG extracted from the raw piezoelectric sensor signal.

Subject	ERB	FRG		
	Respiration acts	Respiration acts	Missed acts	Spurious acts
#1	91	92	0	1
#2	77	78	0	1
#3	87	90	0	3
#4	90	91	0	1
#5	81	81	2	2
#6	101	105	0	4
Total	527	537	2	12

*The missed and spurious acts, are reported for the FRG signal with reference to the acts detected in the ERB signal.*

### Statistical Analyses of Heart Monitoring

In Table 2, the number of heartbeats detected per subject in the ECG and the missed beats identified in the LF-FCG and HF-FCG components (extracted from raw piezoelectric sensor signal) are reported. In particular, a total of 7,316 heartbeats were detected in the ECG, while 376 missed heartbeats were found in the LF-FCG component and 43 in the HF-FCG. Therefore, the LF-FCG and HF-FCG scored a sensitivity of 94.9 and 99.4%, respectively.

**TABLE 2:** Heart beats detected in ECG and in the LF-FCG and HF-FCG components extracted from the raw piezoelectric sensor signal.

Subject	True beats	Missed beats	
	ECG	LF-FCG	HF-FCG
#1	1477	20	12
#2	1404	44	5
#3	884	78	0
#4	1832	127	20
#5	1096	47	6
#6	623	60	0
Total	7316	376	43

*The missed beats are reported with reference to the beats detected in the ECG.*

**FIGURE 8:**
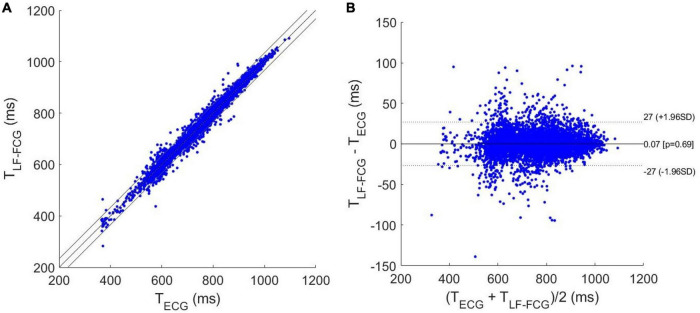
Statistical analyses on inter-beat intervals estimated from ECG and LF-FCG: **(A)** results of regression and correlation analyses; **(B)** results of Bland-Altman analysis.

**FIGURE 9:**
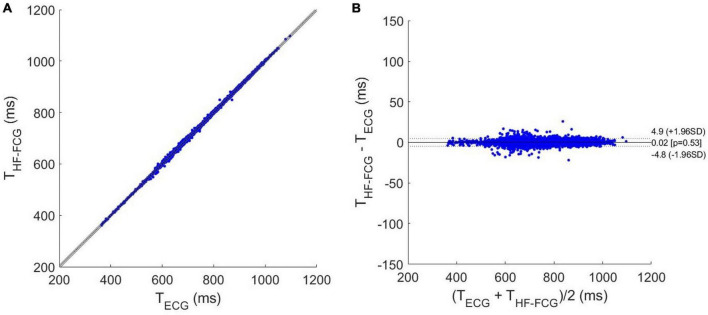
Statistical analyses on inter-beat intervals estimated from ECG and HF-FCG: **(A)** results of regression and correlation analyses; **(B)** results of Bland-Altman analysis.

The results of regression, correlation and Bland-Altman analyses are depicted in Figures 8, 9. The intervals related to missed beats were discarded from the analyses, which were performed on a total of 6,661 inter-beat intervals for LF-FCG and 7,211 for HF-FCG. For LF-FCG, the statistical analyses report a slope and intercept of 1.001 and –0.8 ms (*R*^2^ = 0.986) and a non-significant bias (*p* = 0.69) with limits of agreement of ± 26.9 ms. For HF-FCG, slope and intercept of 1.001 and –0.6 ms (*R*^2^ = 0.999), as well as a non-significant bias (*p* = 0.53) with limits of agreement of ± 4.9 ms were found.

### Comparison With Seismocardiography and Phonocardiography

**FIGURE 10:**
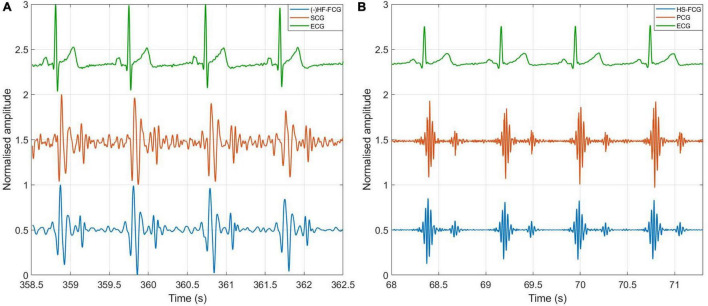
Comparison of FCG with SCG and PCG. **(A)** Example of HF-FCG, SCG and ECG signals acquired simultaneously; **(B)** example of HS-FCG, PCG and ECG signals acquired simultaneously.

Short tracts of HF-FCG and SCG signals, and HS-FCG and PCG signals are shown in Figures 10A,B, respectively.

**FIGURE 11:**
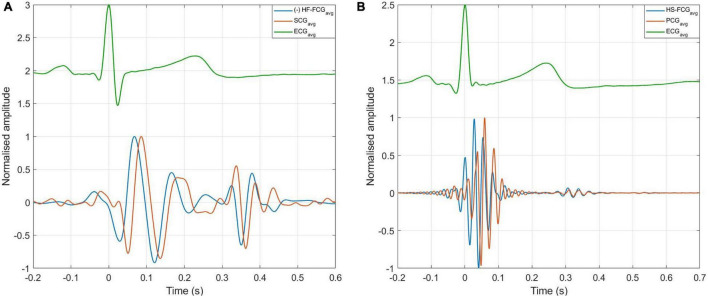
ECG-triggered ensemble averages of FCG, SCG, and PCG. **(A)** Ensemble averages of HF-FCG and SCG; **(B)** ensemble averages of HS-FCG and PCG.

**FIGURE 12:**
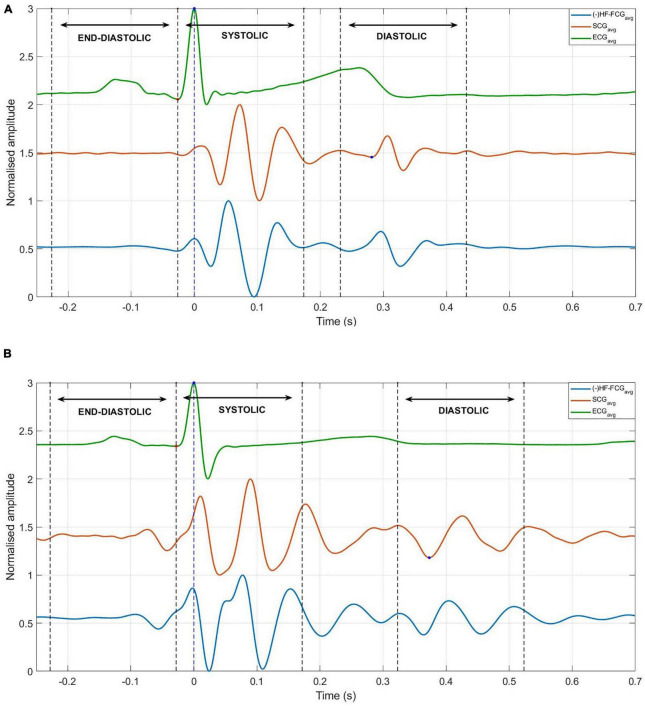
Ensemble averages of HF-FCG, SCG, and ECG, with boundaries of systolic, diastolic, and end-diastolic intervals (dashed lines). **(A)** Example of signals with no significant components in the end-diastolic interval, which scored low correlation indices in the same interval. **(B)** Example of signals with significant components in the end-diastolic interval, which scored high correlation coefficients in the same interval.

An example of the ensemble averages of HF-FCG and SCG signals are shown in Figure 11A, while the ensemble averages of HS-FCG and PCG signals are shown in Figure 11B. In Table 3 the normalized cross-correlation indices between the ensemble averages of HF-FCG vs. SCG and HS-FCG vs. PCG are reported for each subject. Analogously, Table 4 outlines the normalized cross-correlation indices of the ensemble averages of HF-FCG vs. SCG, computed separately in the systolic, diastolic and end-diastolic intervals. The cross-correlation indices scored in the systolic intervals turned out to be similar to those related to the whole cardiac cycle (reported in Table 3); also in the diastolic intervals the ensemble averages scored similar or slightly lower normalized cross-correlation indices. Considerably lower correlations were found in the end-diastolic intervals related to subjects #2, #3, and #5. Indeed, the end-diastolic regions of HF-FCG and SCG ensemble averages of these subjects appeared almost flat with some noisy fluctuations (see Figure 12A), while those of the other subjects exhibited a clear and matching oscillation in both HF-FCG and SCG averages (see Figure 12B). As an example, Figure 12 shows data from subjects #2 and #4, particularly the ensemble averages of HF-FCG, SCG and ECG, with the boundaries of the three considered time intervals.

**TABLE 3:** Cross-correlation indices of HF-FCG vs. SCG and HS-FCG vs. PCG.

Subject	Cross-correlation index	
	HF-FCG vs. SCG	HS-FCG vs. PCG
#1	0.7901	0.8674
#2	0.8939	0.9063
#3	0.8999	0.7816
#4	0.8793	0.7187
#5	0.8559	0.7922
#6	0.8112	0.8357

**TABLE 4:** Cross-correlation indices of HF-FCG vs. SCG in 3 intervals of the cardiac cycle: [ECG-Q; ECG-Q + 200 ms] (systole); [SCG-AC–50 ms; SCG-AC + 150 ms] (diastole); [ECG-Q–200 ms; ECG-Q] (end-diastole).

Subject	Cross-correlation index		
	Systole	Diastole	End-diastole
#1	0.7256	0.6832	0.8187
#2	0.9203	0.8581	0.2985
#3	0.9376	0.7856	0.3555
#4	0.8699	0.8530	0.9265
#5	0.8487	0.6537	0.2672
#6	0.8166	0.8491	0.6253

## Discussion

The raw signals from the proposed PZT sensor and the FSR-based sensor turned out to be very similar, despite some differences in the amplitude ratios of the respiratory and cardiac components. In particular, the FCG signals provided by the two sensors showed up as consistently delayed by about tens of milliseconds and scored Pearson’s correlation coefficients in excess of 0.97 (*p* < 0.05). The statistical analyses showed that the PZT sensor was able to capture, simultaneously, the respiratory acts and the heart beats, and provided accurate measurements of both inter-breath and inter-beat intervals. However, these results must be confirmed by further analyses on a larger cohort of subjects, possibly including also pathological patients. The results prove that, not only the proposed PZT sensor is suitable for simultaneous FRG and FCG measurements, but it also achieves higher performances with respect to the FSR-based one. Indeed, as shown by the much lower LoA of the HF-FCG-derived inter-beat intervals provided by the PZT sensor (during respiration) as compared to those provided by the FSR-based sensor (during apnea, see Andreozzi et al., 2020), the PZT sensor provides more precise and stable measurements and proved capable of acquiring SCG-like (HF-FCG) and heart sounds components with a very high SNR, without the need for ensemble averaging, which is commonly required for SCG signal processing. In addition, with regard to common accelerometers used for SCG studies, they usually exhibit limitations in monitoring movements both at low frequencies, due to the nature of accelerometric measurements, and at high frequencies, due to the impact of noise. This behavior makes it difficult to capture respiration and heart sounds, so the monitoring is usually restricted to the SCG frequency range alone. The proposed piezoelectric FCG sensor, instead, provides accurate, beat-by-beat measurements of chest wall movements in a broad band, thus allowing the simultaneous monitoring of respiration, infrasonic cardiac vibrations and heart sounds with one single device. The comparison with SCG showed that HF-FCG and SCG have a remarkable similarity. Such similarity was found also in the systolic and diastolic intervals of all subjects, while considerably lower normalized cross-correlation indices were found in the end-diastolic intervals (i.e., the interval between two heartbeats) of some subjects. Actually, for these subjects, the signal in the end-diastolic intervals was practically absent, hence the noise prevailed and lowered the cross-correlation index. On the contrary, for the remaining subjects, a clear and matching signal appeared in the end-diastolic interval, resulting in a considerable cross-correlation index. However, these preliminary results do not support the possibility to use the HF-FCG in place of the SCG. Indeed, further analyses on the identification of well-established SCG fiducial markers in HF-FCG are needed to prove its suitability as an alternative to SCG. With regard to the heart sounds, an impressive similarity was observed between the HS-FCG and PCG signals, not only in terms of waveforms morphology, but also in terms of acoustic impression (Supplementary Audio 1). It is important to consider that, due to the noticeable decrease in amplitude from the large FRG signal down to the very tiny heart sounds to be extracted from the digitized raw PZT sensor signal, an high dynamic range is usually demanded for the digitization, so as to ensure, at the same time, a reasonable range for the FRG and a suitable amplitude resolution for the heart sounds. Otherwise, the four components of the raw PZT sensor signal could be split and amplified separately via a proper analog front-end, and then digitized simultaneously as independent signals, by using lower dynamic ranges. However, such an approach would need a careful design, as the FRG component may feature a non-negligible power spectral density at higher frequencies (e.g., due to brisk inspiration/expiration acts, as well as to high respiratory rates) causing artifacts in the resulting FCG signal produced by analog filters. Indeed, precisely for this reason, a Savitzky-Golay smoothing filter was used in this study, which effectively helped to recover many FCG segments that would have been lost by common low-pass filters designed in the frequency domain. Finally, it is worth underlining that the results presented in this study have been obtained via an inexpensive, off-the-shelf piezoelectric transducer. On one hand, this suggests a possible application of the novel FCG sensor also as a disposable device. On the other hand, an optimization of sensor design (e.g., dimensions, shape and material properties of the bare PZT disk and the mechanical coupler) could yield even better performances, such as higher sensibility, higher dynamic range, superior mechanical coupling.

### Limitations of the Study

The data analyzed in this study were acquired on 6 healthy subjects only. Therefore, the preliminary results of the study must be confirmed on a much larger cohort of subjects, possibly including pathological patients.

All the measurements were performed on subjects at rest and comfortably sitting on a chair. The subjects were asked not to breath forcefully, even when intentionally increasing the respiratory rate. The performance of the proposed sensor should be evaluated also on moving subjects and in stress conditions.

The similarity of FCG, SCG and PCG signals was evaluated only based on normalized cross-correlation indices, with no further analysis on specific morphological differences. In addition, it was practically unfeasible to record FCG and PCG from exactly the same point on subjects’ chests.

## Conclusion

This study presented a novel piezoelectric force sensor for simultaneous monitoring of respiration (FRG), infrasonic cardiac vibrations (LF-FCG, HF-FCG) and heart sounds (HS-FCG). The novel sensor was compared to the FSR-based sensor proposed in Andreozzi et al. (2020, 2021) by carrying out measurements on six healthy volunteers, while breathing at rest. The FCG signals provided by the two compared sensors turned out to be very similar, proving that the novel PZT sensor is suitable for FCG. The actual performances of the novel sensor in the estimation of respiratory and heart rates were assessed by comparison with signals from a respiratory band and an ECG monitor, which were acquired simultaneously and assumed as benchmarks. Regression, correlation and Bland-Altman analyses were carried out, which confirmed that the novel piezoelectric FCG sensor provides accurate and precise measurements of both respiratory and heart rates on subjects at rest. In addition, the PZT sensor demonstrated superior performances as compared to the FSR-based one, by providing more accurate and precise measurements, as well as very clear beat-by-beat recordings of both the SCG-like component (HF-FCG) and the heart sounds, without the need to resort to ensemble averaging, as usually required for inertial sensors signals. Finally, the comparison of FCG with dorso-ventral SCG and PCG signals, acquired concurrently via an accelerometer and an electronic stethoscope, revealed that HF-FCG and SCG, as well as HS-FCG and PCG, share a remarkably similar morphology.

To the best of our knowledge, this is the first time in literature that a single piezoelectric sensor in demonstrated for multimodal sensing of cardio-respiratory activity, with the ability to simultaneously capture respiration, heart sounds, an SCG-like signal (HF-FCG) and the LF-FCG signal. As already reported in Andreozzi et al. (2020), the LF-FCG is not visible in SCG recordings and seems to be associated with ventricular emptying and filling events, thus providing additional information that could improve the investigation and comprehension of the mechanical behavior of the beating heart. The possibility to acquire these signals all together with a single piezoelectric sensor suggests that, apart from the Forcerespirogram, which finds its origin in the respiratory activity, the other signals captured by the FCG sensor contain information that represents different aspects of the same event, i.e., the heartbeat, and as such they should be considered as a whole, namely as different components of the same signal: the Forcecardiogram.

In conclusion, the novel piezoelectric FCG sensor here proposed stands as a promising device for accurate, unobtrusive, long-term monitoring of cardiorespiratory functions in subjects at rest and paves the way for a wide spectrum of potential applications, both in the research and clinical fields. As an example, it could ease the data acquisition in studies involving the multimodal monitoring of cardiorespiratory activity, by obviating the need to equip the subjects with multiple instruments, which would improve the overall unobtrusiveness of the measurement setup. Therefore, it could be used in studies aimed at investigating cardiorespiratory interactions (Tang et al., 2017; Radovanović et al., 2018) or in the analysis of sleep disorders, where it can be integrated in polysomnographic instrumentation. The novel FCG sensor could also be used to analyze the cardiac force-frequency relationship in stress tests, to characterize the behavior of failing hearts, such as in Bombardini et al. (2007). From a clinical perspective, the sensor could support the development of several pervasive monitoring applications, such as the very long-term, continuous monitoring of cardiorespiratory functions at home in the elderly and in chronic patients with heart and pulmonary diseases, as well as the discovery of sleep apneas, and other potential telemedicine applications involving cardiorespiratory monitoring. Further studies are foreseen to carry out an in-depth assessment of the relationship between the SCG-like component of FCG (HF-FCG) and the accelerometer-based SCG, with particular focus on the ability to detect well-established SCG fiducial markers (Zakeri et al., 2020). Moreover, the viability of FCG-based monitoring could be also assessed in physical activities, ranging from simple walking to sport activities and execution of heavy works. Finally, the use of FCG sensors matrices could be investigated to gain new insights into the features, distribution, and propagation of heart mechanical vibrations onto the chest wall.

## Data Availability Statement

The datasets presented in this article are not readily available because informed consent from the subjects involved was obtained only for this study and not for public availability. Requests to access the datasets should be directed to PB, paolo.bifulco@unina.it.

## Ethics Statement

Ethical review and approval was not required for the study on human participants in accordance with the local legislation and institutional requirements. The patients/participants provided their written informed consent to participate in this study.

## Author Contributions

EA and PB: conceptualization. EA: methodology, formal analysis, and writing—original draft preparation and visualization. EA, DE, GG, and PB: writing—review and editing and investigation. PB: supervision. All authors have read and agreed to the published version of the manuscript.

## Conflict of Interest

The sensor described in this manuscript is protected by the (pending) patent PCT/AU2020/051107. All the authors are listed as inventors. GG was a minority shareholder of Medical Monitoring Solutions PTY whom owns the mentioned IP.

## Publisher’s Note

All claims expressed in this article are solely those of the authors and do not necessarily represent those of their affiliated organizations, or those of the publisher, the editors and the reviewers. Any product that may be evaluated in this article, or claim that may be made by its manufacturer, is not guaranteed or endorsed by the publisher.
